# Pyridin-1-ium carb­oxy­formate–2-chloro­acetic acid (1/1)

**DOI:** 10.1107/S2414314624012422

**Published:** 2025-01-03

**Authors:** Nurlana D. Sadikhova, Farida M. Muradova, Narmina A. Guliyeva, Khudayar I. Hasanov, Tahir A. Javadzade, Ennio Zangrando, Alebel N. Belay

**Affiliations:** a Department of Chemistry, Baku State University, Z. Khalilov Str. 23, AZ 1148, Baku, Azerbaijan; b Department of Chemical Engineering, Baku Engineering University, Hasan Aliyev, str. 120, Baku, Absheron AZ0101, Azerbaijan; c Western Caspian University, Istiqlaliyyat Street 31, AZ 1001, Baku, Azerbaijan; d Azerbaijan Medical University, Scientific Research Centre (SRC), A. Kasumzade, St. 14. AZ 1022, Baku, Azerbaijan; e Department of Chemistry and Chemical Engineering, Khazar University, Baku, Mahsati st. 41, AZ1096, Baku, Azerbaijan; f Department of Chemical and Pharmaceutical Sciences, University of Trieste, 34127, Trieste, Italy; g Department of Chemistry, Bahir Dar University, PO Box 79, Bahir Dar, Ethiopia

**Keywords:** crystal structure, co-crystal, salt, non-covalent inter­actions, hydrogen-bonding

## Abstract

The asymmetric unit of the title salt co-crystal, C_5_H_6_N^+^·C_2_HO_4_^−^·C_2_H_3_ClO_2_, comprises a pyridinium cation, a carb­oxy­formate anion and a 2-chloro­acetic acid mol­ecule. In the crystal, the components are connected by hydrogen bonds within a one-dimensional chain in the *a*-axis direction which incorporates rather short, charge-assisted O—H⋯O hydrogen bonds; the pyridinium-NH group forms bifurcated N—H⋯(O,O) hydrogen bonds of different strength.

## Abstract 2

A salt co-crystal comprising pyridin-1-ium carb­oxy­formate and 2-chloro­acetic acid is stabilized by conventional and charge-assisted hydrogen bonds within a one-dimensional chain.

## Graphical abstract

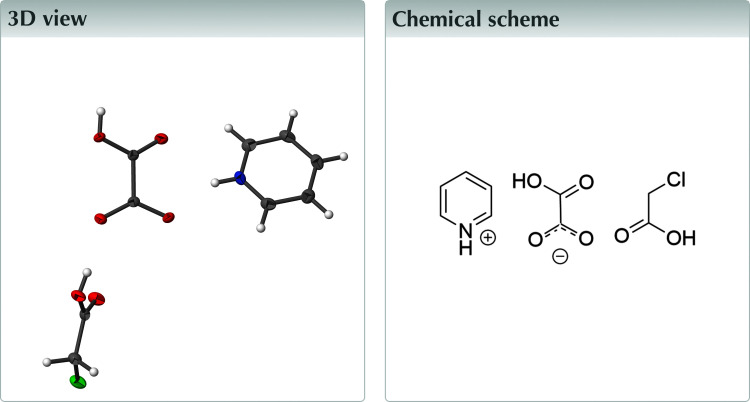

## Structure description

Crystal engineering of co-crystals has inspired great inter­est from researchers in recent times due to their ability to improve functional properties of materials including pharmaceutical active ingredients (Braga *et al.*, 2013 ▸). The selection of synthons or tectons is an important synthetic step to improve the performance of co-crystals, such as solubility, catalytic activity, dissolution profile, pharmacokinetics and stability (Jlassi *et al.*, 2014 ▸; Mahmoudi *et al.*, 2017*a* ▸,*b* ▸). Designing weak intra- or inter­molecular inter­actions and the procedures underlying synthesis constitute the operational part of the crystal engineering endeavour (Abdelhamid *et al.*, 2011 ▸; Afkhami *et al.*, 2017 ▸). The use of various types of non-covalent inter­actions in the design of multi-component co-crystals is based on a complete knowledge of these weak bonds, especially supra­molecular synthons (Berry *et al.*, 2017 ▸; Gurbanov *et al.*, 2018 ▸, 2020 ▸; Kopylovich *et al.*, 2012*a* ▸,*b* ▸).

**Figure 1:**
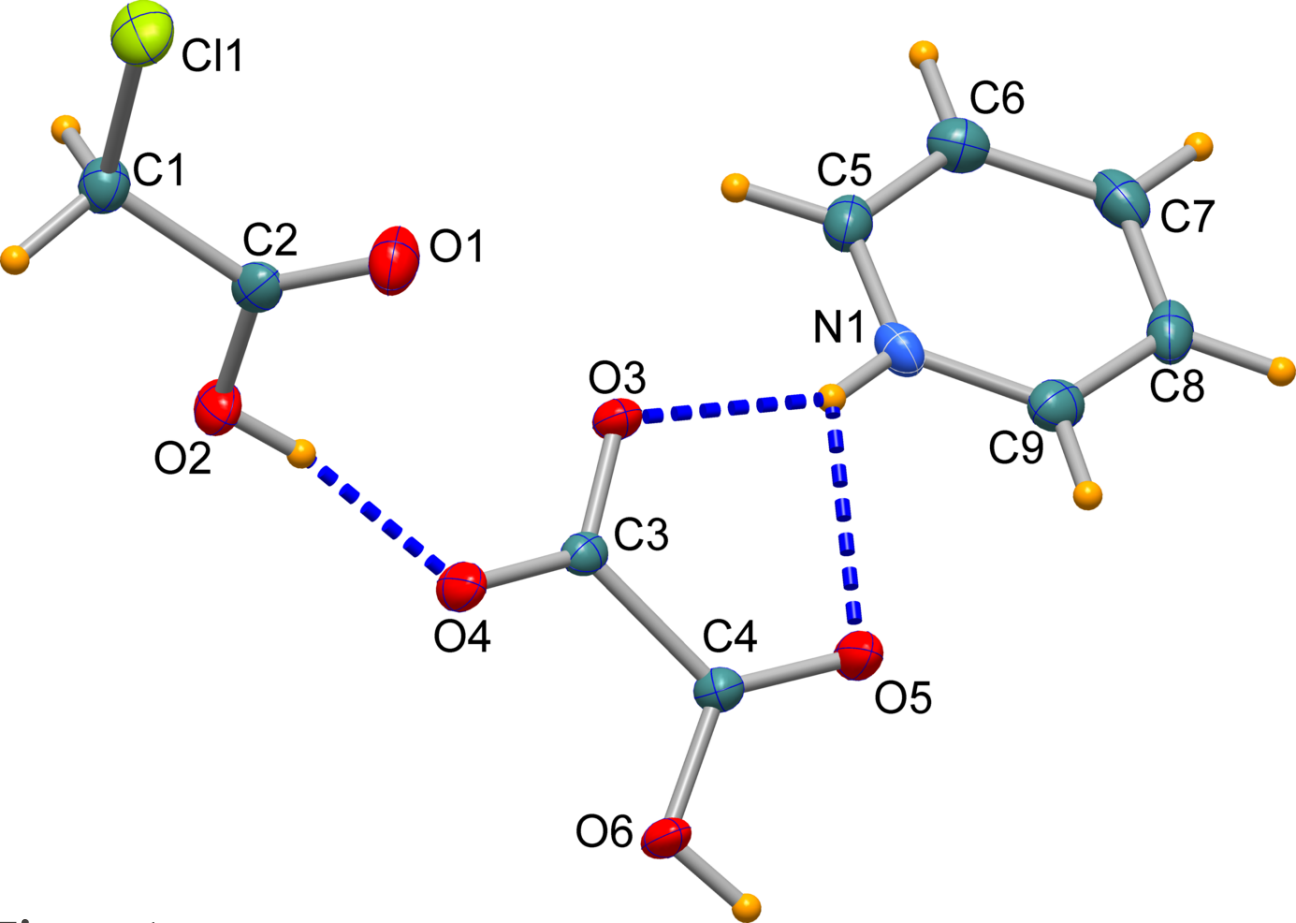
Mol­ecular structures of the components of the asymmetric unit, showing the atom-numbering scheme and displacement ellipsoids at the 50% probability level.

The asymmetric unit of the title compound is shown in Fig. 1 ▸. The pyridinium cation, the carb­oxy­formate anion and the chloro­acetic acid mol­ecule inter­act through hydrogen bonds as described below. The C—OH bond lengths for the chloro­acetic- and oxalate-bound carboxyl­ate groups, of 1.3215 (19) and 1.3073 (17) Å, respectively, are longer in comparison to the C=O bond lengths, which range from 1.2074 (19) Å (chloro­acetic acid) to 1.2653 (17) Å. These observations clearly confirm the positions of the H atoms, which were located in difference-Fourier maps and refined. The C1—Cl1 bond length is 1.7755 (15) Å with this bond being in an eclipsed conformation as the Cl1—C1—C2—O1 torsion angle is −6.4 (2)°. This torsion angle results in a molecular conformation that is close to planar (*C_s_* symmetry) and corresponds to the ground state of the mol­ecule, as confirmed by quantum chemical calculations (Ananyev *et al.*, 2014 ▸).

**Figure 2:**
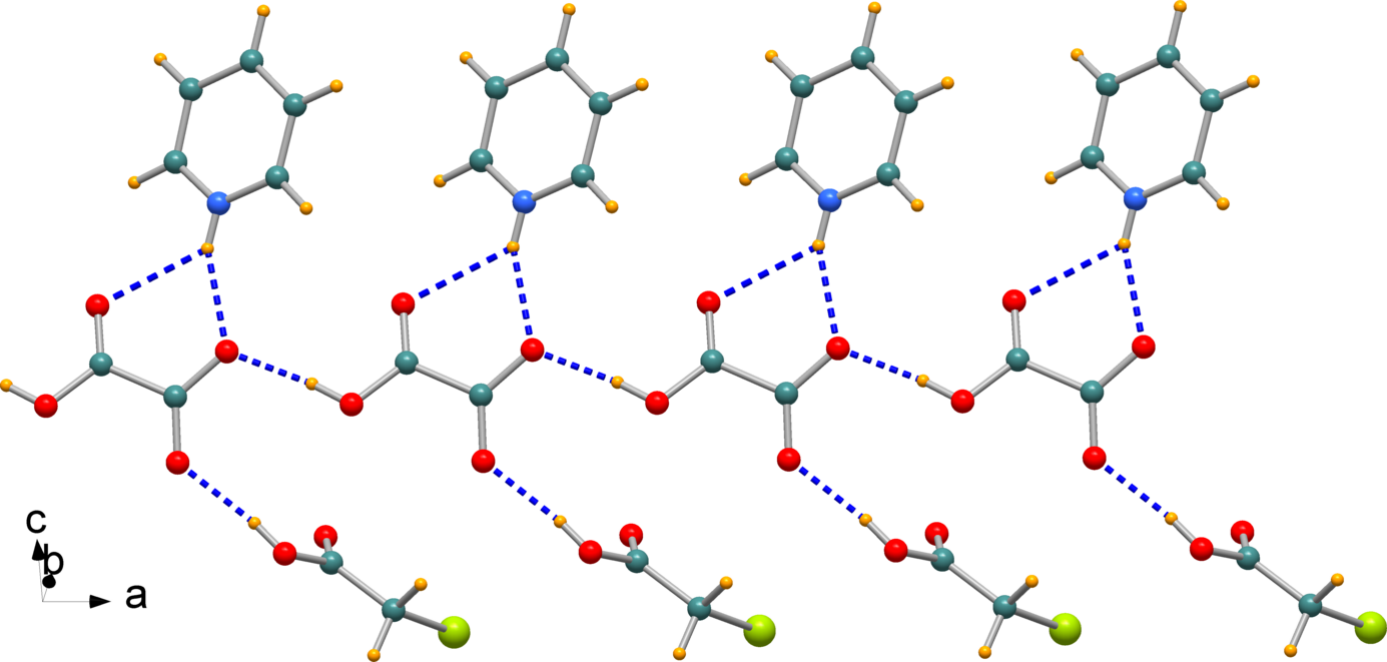
A view of the one-dimensional chain along the *a-*axis direction and featuring hydrogen bonds shown as blue dashed lines.

**Figure 3:**
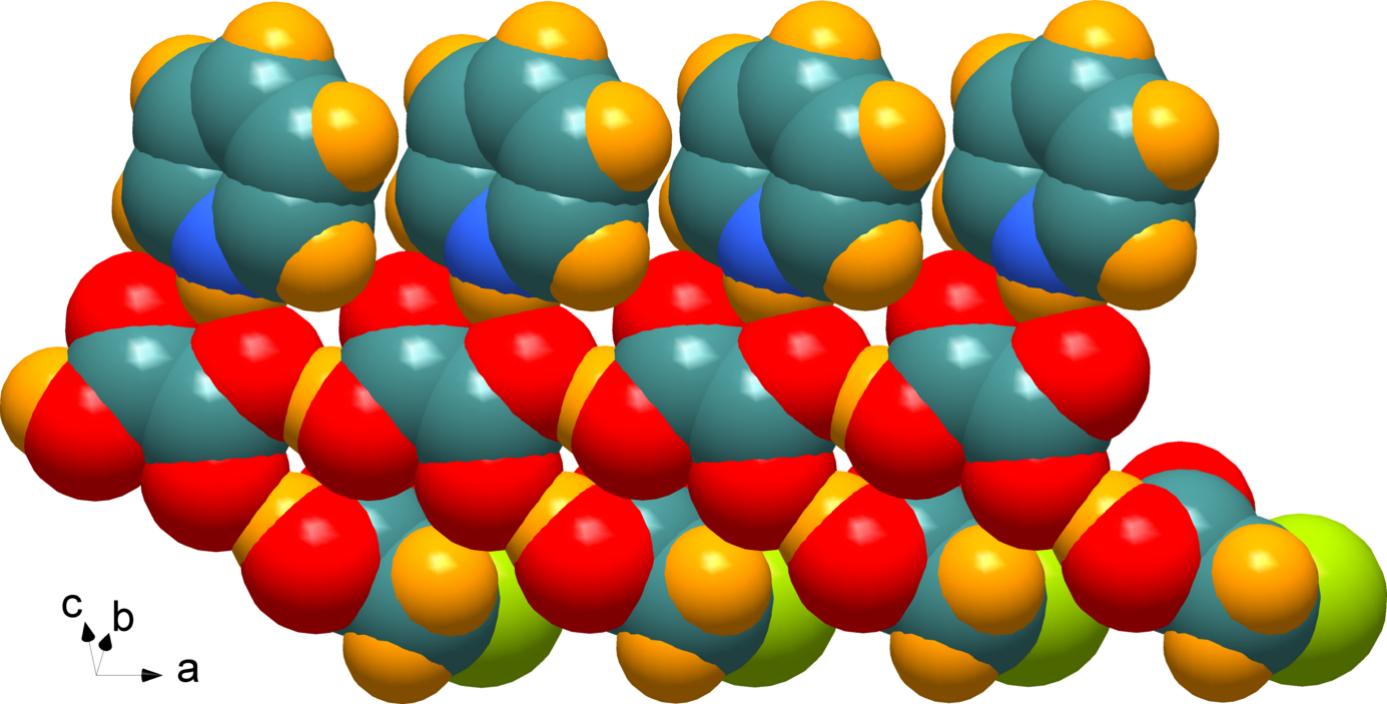
Space-filling representation of the one-dimensional chain.

In the crystal, the species are connected by hydrogen bonds within a linear one-dimensional chain extending along the *a-*axis direction (Fig. 2 ▸); a space-filling representation is displayed in Fig. 3 ▸. The O—H⋯O hydrogen bonds, Table 1 ▸, are rather short with O⋯O separations of 2.5834 (14) and 2.6209 (15) Å, while the pyridinium-NH group forms bifurcated N—H⋯(O,O) hydrogen bonds of different strength, with N⋯O distances of 2.7935 (16) and 2.9546 (17) Å. These ribbons inter­act through non-conventional C—H⋯O hydrogen bonds that consolidate the supra­molecular network, Table 1 ▸. Within the chain, the pyridinium mean plane forms a dihedral angle of 25.92 (6)° with the adjacent carb­oxy­formate anion, which in turn is twisted by 63.73 (4)° with respect to the mean plane through the chloro­acetic acid mol­ecule.

Chloro­acetic acid is a strong carb­oxy­lic acid with p*K*_a_ = 2.7 (Kartrum *et al.*, 1961 ▸). A search of the Cambridge Structural Database (CSD: version 5.45, March 2024; Groom *et al.*, 2016 ▸) retrieved 39 hits containing chloro­acetic acid. Two polymorphs are known, *i.e*. the α- (Kanters & Roelofsen, 1976 ▸) and β-forms (Kanters *et al.*, 1976 ▸). The α-form has two mol­ecules in the asymmetric unit and has been subjected to a variable temperature study, *i.e.* in the range from 90 to 210 K (Ananyev *et al.*, 2014 ▸). This study shows the Cl—C—C=O torsion angles average 22.33 (5) and 1.30 (5)° in the two independent mol­ecules over the temperature range (Ananyev *et al.*, 2014 ▸). Štoček *et al.* (2022 ▸) analysed the position of the H atom in the O—H⋯N hydrogen bond of the structure of chloro­acetic acid with pyridine-4-carboxamide at ten different temperatures. It is also worth noting the crystal structure of quinolinium 2-carboxyl­ate with 2-chloro­acetic acid, a species known to exhibit anti-diabetic activity (Kavitha *et al.*, 2021 ▸).

## Synthesis and crystallization

A mixture of oxalic acid (0.1 mmol), 2-chloro­acetic acid (0.1 mmol) and pyridine (0.1 mmol) in methanol (15 ml) was kept for crystallization. The title compound was obtained as a colourless crystals after 2–3 days, yield 87%.

Analysis calculated for C_9_H_10_ClNO_6_ (*M* = 263.63): C 41.00, H 3.82, N 5.31; Found: C 40.89, H 3.77, N 5.29%. ^1^H NMR (300 MHz, DMSO) *δ* 10.50 (1*H*, NH), 7.45–8.62 (5*H*, py) and 4.27 (2*H*, CH_2_); OH not observed. ^13^C NMR (75 MHz, DMSO) *δ* 41.59, 124.44, 137.53, 148.70, 161.49, 168.72 and 169.23. ESI–MS: m/*z*: 264.58 [*M*+H]^+^.

## Refinement

Crystal data, data collection and structure refinement details are summarized in Table 2 ▸. The H atoms of the carboxyl­ate and pyridinium ions were refined freely.

## Supplementary Material

Crystal structure: contains datablock(s) I. DOI: 10.1107/S2414314624012422/tk4114sup1.cif

Structure factors: contains datablock(s) I. DOI: 10.1107/S2414314624012422/tk4114Isup2.hkl

Supporting information file. DOI: 10.1107/S2414314624012422/tk4114Isup3.cml

CCDC reference: 2412697

Additional supporting information:  crystallographic information; 3D view; checkCIF report

## Figures and Tables

**Table 1:** Hydrogen-bond geometry (Å, °)

*D*—H⋯*A*	*D*—H	H⋯*A*	*D*⋯*A*	*D*—H⋯*A*
O2—H2*o*⋯O4	0.82 (2)	1.81 (2)	2.6209 (15)	170 (2)
O6—H6*o*⋯O3^i^	0.86 (2)	1.72 (2)	2.5834 (14)	171.8 (19)
N1—H1*n*⋯O3	0.861 (19)	1.995 (19)	2.7935 (16)	153.8 (17)
N1—H1*n*⋯O5	0.861 (19)	2.339 (18)	2.9546 (17)	128.7 (15)
C1—H1*a*⋯O1^ii^	0.99	2.54	3.4555 (19)	153
C1—H1*b*⋯O4^iii^	0.99	2.56	3.3439 (19)	136
C5—H5⋯O6^iv^	0.95	2.59	3.3946 (18)	142
C8—H8⋯O1^v^	0.95	2.40	3.3400 (19)	172
C9—H9⋯O5	0.95	2.49	3.0356 (19)	116

Symmetry codes: (i) 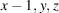; (ii) 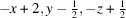; (iii) 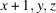; (iv) 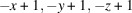; (v) 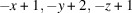.

**Table 2:** Experimental details

Crystal data	
Chemical formula	C_5_H_6_N^+^·C_2_HO_4_^−^·C_2_H_3_ClO_2_
*M*_r_	263.63
Crystal system, space group	Monoclinic, *P*2_1_/*c*
Temperature (K)	150
*a*, *b*, *c* (Å)	5.6911 (4), 7.9862 (4), 24.9303 (14)
β (°)	91.008 (3)
*V* (Å^3^)	1132.91 (12)
*Z*	4
Radiation type	Mo *K*α
μ (mm^−1^)	0.35
Crystal size (mm)	0.37 × 0.26 × 0.15

Data collection	
Diffractometer	Bruker APEXII CCD
Absorption correction	Multi-scan (*SADABS*; Krause *et al.*, 2015 ▸)
*T*_min_, *T*_max_	0.872, 0.939
No. of measured, independent and observed [*I* > 2σ(*I*)] reflections	7091, 2127, 1963
*R*_int_	0.030
(sin θ/λ)_max_ (Å^−1^)	0.611

Refinement	
*R*[*F*^2^ > 2σ(*F*^2^)], *wR*(*F*^2^), *S*	0.031, 0.082, 1.10
No. of reflections	2127
No. of parameters	163
H-atom treatment	H atoms treated by a mixture of independent and constrained refinement
Δρ_max_, Δρ_min_ (e Å^−3^)	0.19, −0.32

Computer programs: *APEX4* and *SAINT* (Bruker, 2012 ▸), *SHELXT* (Sheldrick, 2015*a* ▸), *SHELXL* (Sheldrick, 2015*b* ▸) and *DIAMOND* Brandenburg, 1999 ▸).

## full crystallographic data

### Pyridin-1-ium carboxyformate–2-chloroacetic acid (1/1) Crystal data

**Table:**

C_5_H_6_N^+^·C_2_HO_4_^−^·C_2_H_3_ClO_2_	*F*(000) = 544
*M**_r_* = 263.63	*D*_x_ = 1.546 Mg m^−^^3^
Monoclinic, *P*2_1_/*c*	Mo *K*α radiation, λ = 0.71073 Å
*a* = 5.6911 (4) Å	Cell parameters from 4685 reflections
*b* = 7.9862 (4) Å	θ = 2.7–25.7°
*c* = 24.9303 (14) Å	µ = 0.35 mm^−^^1^
β = 91.008 (3)°	*T* = 150 K
*V* = 1132.91 (12) Å^3^	Plate, colourless
*Z* = 4	0.37 × 0.26 × 0.15 mm

### Pyridin-1-ium carboxyformate–2-chloroacetic acid (1/1) Data collection

**Table:**

Bruker APEXII CCD diffractometer	1963 reflections with *I* > 2σ(*I*)
φ and ω scans	*R*_int_ = 0.030
Absorption correction: multi-scan (SADABS; Krause *et al.*, 2015)	θ_max_ = 25.7°, θ_min_ = 2.7°
*T*_min_ = 0.872, *T*_max_ = 0.939	*h* = −4→6
7091 measured reflections	*k* = −9→9
2127 independent reflections	*l* = −30→29

### Pyridin-1-ium carboxyformate–2-chloroacetic acid (1/1) Refinement

**Table:**

Refinement on *F*^2^	Primary atom site location: structure-invariant direct methods
Least-squares matrix: full	Secondary atom site location: difference Fourier map
*R*[*F*^2^ > 2σ(*F*^2^)] = 0.031	Hydrogen site location: mixed
*wR*(*F*^2^) = 0.082	H atoms treated by a mixture of independent and constrained refinement
*S* = 1.10	*w* = 1/[σ^2^(*F*_o_^2^) + (0.0367*P*)^2^ + 0.480*P*] where *P* = (*F*_o_^2^ + 2*F*_c_^2^)/3
2127 reflections	(Δ/σ)_max_ = 0.001
163 parameters	Δρ_max_ = 0.19 e Å^−^^3^
0 restraints	Δρ_min_ = −0.32 e Å^−^^3^

### Pyridin-1-ium carboxyformate–2-chloroacetic acid (1/1) Special details

**Table:**

Geometry. All esds (except the esd in the dihedral angle between two l.s. planes) are estimated using the full covariance matrix. The cell esds are taken into account individually in the estimation of esds in distances, angles and torsion angles; correlations between esds in cell parameters are only used when they are defined by crystal symmetry. An approximate (isotropic) treatment of cell esds is used for estimating esds involving l.s. planes.

### Pyridin-1-ium carboxyformate–2-chloroacetic acid (1/1) Fractional atomic coordinates and isotropic or equivalent isotropic displacement parameters (Å^2^)

**Table:**

	*x*	*y*	*z*	*U*_iso_*/*U*_eq_	
Cl1	1.30381 (7)	0.60040 (5)	0.25074 (2)	0.02973 (14)	
O1	0.8928 (2)	0.68016 (13)	0.31709 (5)	0.0274 (3)	
O2	0.8175 (2)	0.41486 (13)	0.34182 (4)	0.0219 (3)	
H2o	0.717 (4)	0.457 (2)	0.3605 (8)	0.033*	
O3	0.62947 (17)	0.65703 (12)	0.47108 (4)	0.0177 (2)	
O4	0.47024 (18)	0.51382 (13)	0.40178 (4)	0.0202 (2)	
O5	0.19134 (18)	0.77629 (14)	0.49141 (5)	0.0259 (3)	
O6	0.04269 (18)	0.56931 (13)	0.44029 (4)	0.0193 (2)	
H6o	−0.090 (4)	0.608 (2)	0.4516 (8)	0.029*	
N1	0.5976 (2)	0.86111 (15)	0.56167 (5)	0.0196 (3)	
H1n	0.561 (3)	0.807 (2)	0.5329 (8)	0.024*	
C1	1.1476 (3)	0.45380 (19)	0.29004 (6)	0.0211 (3)	
H1a	1.091788	0.361048	0.266754	0.025*	
H1b	1.255256	0.405946	0.317640	0.025*	
C2	0.9388 (3)	0.53258 (18)	0.31738 (6)	0.0183 (3)	
C3	0.4606 (2)	0.60253 (16)	0.44239 (6)	0.0146 (3)	
C4	0.2140 (2)	0.65877 (17)	0.46095 (5)	0.0152 (3)	
C5	0.8036 (3)	0.83223 (19)	0.58675 (6)	0.0222 (3)	
H5	0.908910	0.750929	0.573162	0.027*	
C6	0.8617 (3)	0.92126 (19)	0.63237 (6)	0.0244 (3)	
H6	1.007643	0.902500	0.650458	0.029*	
C7	0.7051 (3)	1.03825 (19)	0.65155 (6)	0.0245 (4)	
H7	0.743544	1.100904	0.682892	0.029*	
C8	0.4919 (3)	1.06414 (18)	0.62502 (6)	0.0236 (3)	
H8	0.382242	1.143181	0.638176	0.028*	
C9	0.4420 (3)	0.97347 (18)	0.57938 (6)	0.0221 (3)	
H9	0.297675	0.990351	0.560428	0.027*	

### Pyridin-1-ium carboxyformate–2-chloroacetic acid (1/1) Atomic displacement parameters (Å^2^)

**Table:**

	*U*^11^	*U*^22^	*U*^33^	*U*^12^	*U*^13^	*U*^23^
Cl1	0.0322 (3)	0.0305 (2)	0.0269 (2)	−0.00243 (17)	0.01274 (18)	−0.00068 (16)
O1	0.0301 (6)	0.0210 (6)	0.0315 (6)	0.0058 (5)	0.0080 (5)	−0.0001 (5)
O2	0.0218 (6)	0.0212 (5)	0.0230 (6)	0.0007 (4)	0.0081 (5)	−0.0047 (4)
O3	0.0113 (5)	0.0204 (5)	0.0212 (5)	0.0000 (4)	−0.0005 (4)	−0.0032 (4)
O4	0.0167 (5)	0.0245 (5)	0.0197 (5)	−0.0009 (4)	0.0043 (4)	−0.0068 (4)
O5	0.0165 (6)	0.0284 (6)	0.0329 (6)	−0.0011 (5)	0.0039 (5)	−0.0153 (5)
O6	0.0100 (5)	0.0251 (5)	0.0229 (6)	−0.0001 (4)	0.0002 (4)	−0.0077 (4)
N1	0.0252 (7)	0.0165 (6)	0.0173 (6)	−0.0043 (5)	0.0025 (5)	−0.0038 (5)
C1	0.0218 (8)	0.0212 (7)	0.0203 (7)	0.0014 (6)	0.0034 (6)	−0.0013 (6)
C2	0.0179 (7)	0.0218 (7)	0.0150 (7)	0.0001 (6)	−0.0013 (6)	−0.0032 (6)
C3	0.0138 (7)	0.0139 (6)	0.0160 (7)	−0.0003 (5)	0.0015 (6)	0.0025 (5)
C4	0.0138 (7)	0.0177 (7)	0.0140 (7)	−0.0004 (5)	0.0002 (5)	0.0008 (5)
C5	0.0220 (8)	0.0182 (7)	0.0265 (8)	0.0016 (6)	0.0055 (6)	−0.0003 (6)
C6	0.0230 (8)	0.0250 (8)	0.0251 (8)	−0.0032 (6)	−0.0014 (7)	0.0009 (6)
C7	0.0336 (9)	0.0203 (7)	0.0198 (8)	−0.0076 (7)	0.0045 (7)	−0.0035 (6)
C8	0.0288 (9)	0.0158 (7)	0.0264 (8)	0.0018 (6)	0.0107 (7)	0.0000 (6)
C9	0.0200 (8)	0.0194 (7)	0.0271 (8)	−0.0009 (6)	0.0011 (6)	0.0035 (6)

### Pyridin-1-ium carboxyformate–2-chloroacetic acid (1/1) Geometric parameters (Å, º)

**Table:**

Cl1—C1	1.7755 (15)	C1—H1a	0.9900
O1—C2	1.2074 (19)	C1—H1b	0.9900
O2—C2	1.3215 (19)	C3—C4	1.551 (2)
O2—H2o	0.82 (2)	C5—C6	1.377 (2)
O3—C3	1.2653 (17)	C5—H5	0.9500
O4—C3	1.2377 (17)	C6—C7	1.382 (2)
O5—C4	1.2155 (18)	C6—H6	0.9500
O6—C4	1.3073 (17)	C7—C8	1.388 (2)
O6—H6o	0.86 (2)	C7—H7	0.9500
N1—C5	1.339 (2)	C8—C9	1.374 (2)
N1—C9	1.341 (2)	C8—H8	0.9500
N1—H1n	0.861 (19)	C9—H9	0.9500
C1—C2	1.517 (2)		
			
C2—O2—H2o	110.2 (14)	O5—C4—C3	121.09 (13)
C4—O6—H6o	109.1 (13)	O6—C4—C3	113.36 (12)
C5—N1—C9	122.63 (14)	N1—C5—C6	119.49 (14)
C5—N1—H1n	120.0 (12)	N1—C5—H5	120.3
C9—N1—H1n	117.3 (12)	C6—C5—H5	120.3
C2—C1—Cl1	112.23 (10)	C5—C6—C7	119.22 (15)
C2—C1—H1a	109.2	C5—C6—H6	120.4
Cl1—C1—H1a	109.2	C7—C6—H6	120.4
C2—C1—H1b	109.2	C6—C7—C8	119.97 (14)
Cl1—C1—H1b	109.2	C6—C7—H7	120.0
H1a—C1—H1b	107.9	C8—C7—H7	120.0
O1—C2—O2	125.61 (14)	C9—C8—C7	118.84 (14)
O1—C2—C1	125.02 (14)	C9—C8—H8	120.6
O2—C2—C1	109.37 (12)	C7—C8—H8	120.6
O4—C3—O3	127.95 (13)	N1—C9—C8	119.83 (15)
O4—C3—C4	117.59 (12)	N1—C9—H9	120.1
O3—C3—C4	114.44 (12)	C8—C9—H9	120.1
O5—C4—O6	125.55 (13)		
			
Cl1—C1—C2—O1	−6.4 (2)	C9—N1—C5—C6	−0.5 (2)
Cl1—C1—C2—O2	174.23 (10)	N1—C5—C6—C7	0.4 (2)
O4—C3—C4—O5	−161.69 (14)	C5—C6—C7—C8	0.3 (2)
O3—C3—C4—O5	17.27 (19)	C6—C7—C8—C9	−0.9 (2)
O4—C3—C4—O6	17.70 (18)	C5—N1—C9—C8	0.0 (2)
O3—C3—C4—O6	−163.35 (12)	C7—C8—C9—N1	0.7 (2)

### Pyridin-1-ium carboxyformate–2-chloroacetic acid (1/1) Hydrogen-bond geometry (Å, º)

**Table:**

*D*—H···*A*	*D*—H	H···*A*	*D*···*A*	*D*—H···*A*
O2—H2*o*···O4	0.82 (2)	1.81 (2)	2.6209 (15)	170 (2)
O6—H6*o*···O3^i^	0.86 (2)	1.72 (2)	2.5834 (14)	171.8 (19)
N1—H1*n*···O3	0.861 (19)	1.995 (19)	2.7935 (16)	153.8 (17)
N1—H1*n*···O5	0.861 (19)	2.339 (18)	2.9546 (17)	128.7 (15)
C1—H1*a*···O1^ii^	0.99	2.54	3.4555 (19)	153
C1—H1*b*···O4^iii^	0.99	2.56	3.3439 (19)	136
C5—H5···O6^iv^	0.95	2.59	3.3946 (18)	142
C8—H8···O1^v^	0.95	2.40	3.3400 (19)	172
C9—H9···O5	0.95	2.49	3.0356 (19)	116

Symmetry codes: (i) *x*−1, *y*, *z*; (ii) −*x*+2, *y*−1/2, −*z*+1/2; (iii) *x*+1, *y*, *z*; (iv) −*x*+1, −*y*+1, −*z*+1; (v) −*x*+1, −*y*+2, −*z*+1.
